# Integrating COVID-19 Vaccination Into Primary Health Care as an Opportunity to Leverage Investments and Build a More Resilient Health System

**DOI:** 10.9745/GHSP-D-23-00420

**Published:** 2024-02-20

**Authors:** Jackline Kiarie, Edward Oladele, Gretchen De Silva, Erica Nybro, Irina Yacobson

**Affiliations:** a Amref Health Africa, Nairobi, Kenya.; b FHI 360, Lusaka, Zambia.; c Bureau for Global Health, U.S. Agency for International Development, Washington, DC, USA.; d Johns Hopkins Center for Communication Programs, Baltimore, MD, USA.; e FHI 360, Durham, NC, USA.

## INTRODUCTION

In May 2023, the World Health Organization (WHO) declared that although COVID-19 was no longer a global public health emergency, the pandemic was not over and would require long-term management. With that shift, efforts have moved away from stand-alone mass vaccination campaigns and toward interventions that integrate COVID-19 vaccination into life course vaccination programs for at-risk populations within the primary health care (PHC) system.^1^ To assist countries with this shift, WHO published programmatic considerations for integrating COVID-19 vaccination into PHC and immunization programs, defining integration as^2^:

*The partial or full adoption of COVID-19 vaccination into national immunization programme services, PHC and any other relevant health services with the overall aim of improving programme efficiency and sustainability, enhancing demand and improving user satisfaction, achieving and maintaining satisfactory coverage, and addressing inequities.*

The U.S. Agency for International Development (USAID) builds upon this definition to include not only vaccination but also COVID-19 response activities related to diagnosis, care, and treatment.^3^

Although this global guidance exists, it is not intended to be prescriptive. As implementing partners and governments reflect on this guidance and the new phase of COVID-19 management in their respective countries, many questions remain on how best to operationalize integration and incorporate COVID-19-related response activities within their complex health system environments. We do not assume that COVID-19 vaccination will and should always be a top priority compared to other needed health interventions. Integration of any new service has costs. Resources are finite, and funds, human resources, supplies, and time that go toward COVID-19 vaccination divert those resources away from other essential health care areas.

As country priorities shift and donor funds for COVID-19 response efforts decrease, integration of COVID-19 vaccination into routine health service provision has the potential to streamline the delivery of health care interventions and realize health workforce and cost efficiencies that are not available when health care service delivery is siloed. There are opportunities for integration at different national and subnational levels. Within these diverse national and subnational contexts, stakeholders at all levels should consider opportunities for integration, especially where resources are limited. In some settings, stakeholders have prioritized integration at service delivery sites where COVID-19 vaccinations are provided alongside routine immunizations or other health services. However, the health system is far more complex than just service delivery. WHO and USAID note that COVID-19 integration can benefit other critical health system pillars, such as leadership and governance, health systems financing, health workforce, demand generation and community engagement, health information systems, and supply chain management.

## CONSOLIDATING EVIDENCE FROM COVID-19 VACCINE INTEGRATION EFFORTS IN PRIMARY HEALTH CARE

This supplement seeks to contribute to the body of evidence on successful and promising efforts to integrate the COVID-19 vaccination into PHC services and systems in low- and middle-income countries (LMICs). We selected articles based on the quality of the evidence and programmatic relevance of the integration example and directionality of the integration activity (meaning integrating COVID-19 vaccination into existing PHC services and not integrating other health care services into mass COVID-19 vaccination campaigns). In addition, we selected articles that offered a forward-looking perspective that looked at integration beyond the COVID-19 pandemic response efforts and at sustainable integration into other health care services. Upon review of the submitted articles, we noted the following observations.

Due to the recency of the availability of COVID-19 vaccines in much of the world (March 2022) and integration guidance more specifically (May 2023), large-scale studies on the effectiveness of integration efforts are limited, and the longer-term impact and sustainability of such activities cannot yet be measured. Nevertheless, policymakers and program implementers require evidence to inform how to adapt vaccination programming efforts to their context-specific and population needs.

Although efforts have shifted from mass vaccination campaigns to focusing on vaccination of high-priority groups, such as people living with HIV,^4^ as recommended in the WHO SAGE Roadmap guidance,^5^ few of the submissions received focused on integration activities that vaccinate the highest-priority populations. Many submissions highlighted integration in routine immunization and child health services as the most common approach to integration, but that approach may not be sufficient to reach those most at risk unless and until a life course immunization approach becomes the norm.

Although one of the criteria for inclusion in this supplement was the relevance and applicability to other settings, we acknowledge that the impact of the COVID-19 pandemic, the priorities of national and local governments, and plans for COVID-19 integration into PHC are highly context specific and dynamic. There is no single model that will work in all settings or at all times. The articles in this supplement offer country-level experiences of integration but also discuss the practical challenges faced during the integration process. We hope that the selected articles, while appropriately specific to each context, may be useful in other settings.

## A SAMPLE OF PROMISING INTEGRATION EFFORTS

The articles in this supplement, which span all 7 key areas identified by WHO,^2^ highlight promising approaches to the integration of the COVID-19 vaccine into PHC from 17 LMICs in Africa and 2 LMICs in Asia.

In their assessment of ongoing integration interventions in 11 sub-Saharan African countries, Mirza et al.^6^ summarize the facilitators, challenges, and lessons learned, making an argument for the opportunities that integration presents to leverage the COVID-19 response investments to strengthen PHC systems. These investments can pave the way for resilient life course vaccination programs and help systems prepare for future health emergencies.

Tibbels et al.^7^ share the results of qualitative research in Malawi to improve understanding of vaccine hesitancy and misinformation about the COVID-19 and cholera vaccines, contributing information about how individuals conceptualize and make decisions about adult vaccination. This information can help inform strategies to integrate demand creation and delivery of the COVID-19 vaccine with other adult vaccinations and/or disease responses in similar settings.

In their article on integrating COVID-19 vaccination into the existing polio vaccination infrastructure in South Sudan, Kisanga et al.^8^ present the advantages of this approach, which reached more than 700,000 individuals with COVID-19 vaccines while maintaining or increasing polio and other routine immunization coverage.

Mokaya et al.^9^ detail interventions in South Sudan and Sierra Leone that demonstrate the importance of integrated COVID-19 and routine immunization services, particularly in fragile settings. These interventions include an integrated COVID-19 and measles response in Sierra Leone and a data dashboard and health workforce support for combining routine immunization and COVID-19 vaccination delivery in South Sudan.

To address COVID-19 data management challenges in Nigeria, an Electronic Management of Immunization Data system was developed and designed to integrate with the DHIS2 platform to facilitate the management of all immunization and other PHC service data. Tella-Lah et al.^10^ describe the user challenges encountered and the process of optimizing the system that has proven successful in triangulating COVID-19 and other routine immunization data and has the potential for future expansion.

Lava et al.^11^ share the process of adding COVID-19 screening and vaccination services into PHC services that were funded by the national insurance program in the Philippines. They highlight key considerations in the integration of adult vaccination into the care benefit package, barriers, and its implications in access to care during health emergencies.

Monitoring adverse events following immunization is key to ensuring public safety throughout immunization activities and contributes to demand and confidence in vaccines. Hagos et al.^12^ describe an intervention to strengthen the adverse events following immunization surveillance systems in Pakistan and Ethiopia.

Akhlaghi et al.^13^ document the application of the Vaccine Collaborative Supply Planning (VCSP) Initiative during the COVID-19 pandemic to ensure equitable vaccine supply and distribution to where it was most needed. In 15 countries in sub-Saharan Africa, the VCSP Initiative worked to increase vaccine data quality and visibility to help forecast demand and inform supply chain decisions. The authors present the benefits and challenges of the VCSP system that may inform future strengthening of forecasting and supply planning more broadly.

## CONCLUSION

As the global community shifts away from an emergency response to COVID-19 and related large-scale vaccination campaigns, COVID-19 vaccines should remain an available and accessible option, especially for high-priority populations, such as older adults, people with relevant comorbidities (including younger adults), people with immunocompromising conditions (e.g., people living with HIV), pregnant persons, and health workers. One method of accomplishing this is to integrate COVID-19 vaccination into PHC as part of a life course vaccination model. Articles in this supplement provide real-world experiences of how integration has happened and detail potential challenges to prepare for.^6^^,^^8^^,^^9^^,^^11^ Integration through a life course vaccination approach depends on the demand for adult vaccines, or at least, a reduction of vaccine hesitancy and management of vaccine-related disinformation in an integrated system as key elements of generating demand for life course vaccination.^7^

The enormous amount of resources invested in supporting the COVID-19 pandemic response provided opportunities for broader health system strengthening, both for standard care and as a means of preventing and preparing for future public health emergencies, such as the examples seen in the work of Akhlaghi et al., Hagos et al., and Tella-Lah et al.^10^^,^^12^^,^^13^ Lessons learned from the COVID-19 pandemic during the emergency phase and, more recently, through the integration of vaccination activities into PHC, such as those shared in this supplement, should be used to inform policymakers, researchers, and program managers as they strive to consider ways to build resilient health systems while integrating health care services, such as COVID-19 vaccination, into the broader immunization and PHC system.
